# Environmental and genetic determinants of sensorimotor asymmetries in mother-infant interaction

**DOI:** 10.3389/fnbeh.2022.1080141

**Published:** 2022-12-05

**Authors:** Gianluca Malatesta, Daniele Marzoli, Luca Tommasi

**Affiliations:** Department of Psychological, Health and Territorial Sciences, University ‘G. d'Annunzio' of Chieti-Pescara, Chieti, Italy

**Keywords:** functional lateralization, brain organization, maternal effects, cradling-side bias, atypical development

## Introduction

Much evidence indicates that atypical cerebral and/or behavioral lateralization is related to several physical and psychological conditions such as language deficits (Monjauze et al., 2011; Mundorf et al., 2021), autism (Forrester et al., 2014), schizophrenia (Caligiuri et al., 2005), and many others (e.g., see Mundorf and Ocklenburg, 2021; Berretz and Packheiser, 2022). The most noticeable instance of population-level lateralized behavior in humans is right-handedness (i.e., about 90% of humans show a right-hand preference for different manual tasks; Papadatou-Pastou et al., 2020), which is why its systematic deviation, left-handedness, has been extensively investigated in relation to several deficits (e.g., in cognitive abilities such as intelligence and spatial abilities; Gibson, 1973; Johnston et al., 2009; Nicholls et al., 2010; Papadatou-Pastou and Tomprou, 2015; Somers et al., 2015; Piro, 1998). Moreover, left-handedness has been considered as a cue of reduced fitness (in terms of ability to survive and being fit in a given environment; Coren and Halpern, 1991; Deary et al., 2007; Strenze, 2007), along with other negative predictors of fitness (e.g., fluctuating asymmetries such as those of ear, digit, or wrist) that seem to be linked with atypical brain asymmetries (Thoma et al., 2002) and left-handedness itself (Kobyliansky and Micle, 1986). These findings are consistent with the hypothesis that atypical cerebral and/or behavioral lateralization might reflect a potentially dysfunctional brain organization, maybe due to a non-optimal distribution across the two hemispheres of specific functions. In fact, although the scientific debate on typical brain lateralization is still ongoing, the prototypical brain template of lateralized functions posits the left hemisphere as more dominant for limb motor control, language and calculation, and the right hemisphere as more dominant for spatial abilities and emotion recognition from faces and speech (see Vingerhoets, 2019; Forrester et al., 2020; Pfeifer et al., 2022). Given that left-handers (and also mixed-handers; Corballis et al., 2008) often show a hemispheric shift from left to right for language dominance and limb motor control, according to the “cognitive crowding hypothesis,” the disadvantages exhibited by individuals showing atypically-lateralized (but not necessarily reversed) templates might be due to the fierce competition in which such functions are permanently engaged with the other cognitive functions typically located in the other hemisphere (McManus, 2002; Lidzba et al., 2006; Nicholls et al., 2010; Papadatou-Pastou and Tomprou, 2015). Therefore, the putative negative traits associated with left-handedness might not be due to left-handedness *per se*, but rather to an increased chance of cognitive crowding.

## Asymmetry in mother-infant interaction: The left-cradling bias

**Figure 1:**
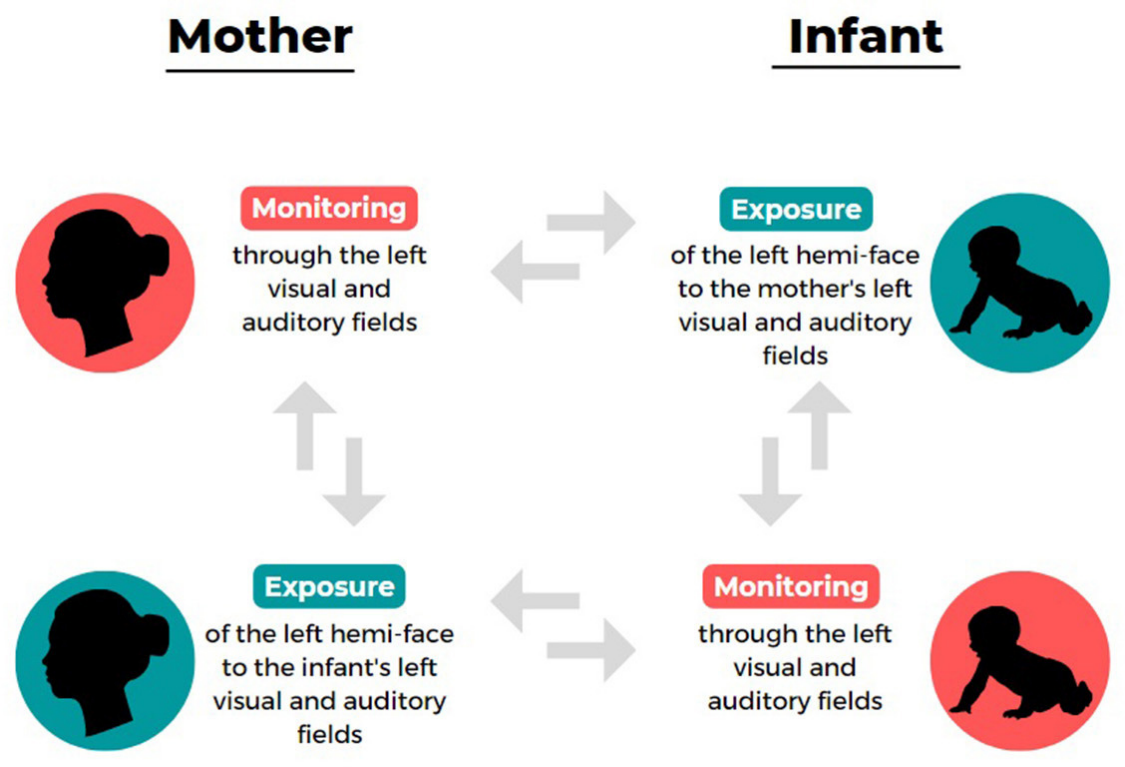
The double exchange system of left-cradling bias (LCB) “monitoring and exposure” functions from the perspective of mother and infant.

It should be said that handedness is not the only instance of strong population-level motor asymmetry at the center of a great deal of research. As an example, footedness (whose prevalence is similar to that of handedness; Porac and Coren, 1981; Tran et al., 2014) has also been extensively investigated. Even sensory asymmetries such as eyedness and earedness, which are less easily observable but whose population-level degree of asymmetries is rather significant (70% for right-eyedness; 60% for right-earedeness; Porac and Coren, 1981; Saudino and McManus, 1998; Tran et al., 2014) have been largely studied over the years in terms of advantages and disadvantages of cerebral lateralization. However, only recently another population-level asymmetry (whose nature likely entails both motor and sensory dimensions) has gained increasing attention: the left-cradling bias (LCB), namely the tendency (observed in over 65% of women), fairly stable across ontogeny, to hold an infant on the left side of their own body during non-functional interactions (Salk, 1960; Sieratzki and Woll, 2002). Recent meta-analyses showed that this asymmetry is observed almost independently of the cradlers' handedness, with left-handers also showing a significant left-side bias, albeit to a lesser extent compared with right-handers (Packheiser et al., 2019). Several adaptive explanations have been suggested for such a bias, but the one receiving more empirical support is the “right-hemisphere hypothesis,” involving that cradling a baby on the left would facilitate the early postnatal communication of socio-emotional information through the right hemispheres of both the cradling and the cradled individuals, which are engaged in a sort of double exchange system (Manning and Chamberlain, 1991; Harris et al., 2001; Bourne and Todd, 2004; Vauclair, 2022; see Giljov et al., 2018 for similar considerations in non-human species). This means that cradling motor behavior might represent a specific interactional “monitoring and exposure” system (see Figure 1) which benefits both the mother and the infant and was presumably shaped by evolutionary and social pressures. In detail, the LCB seems to facilitate the monitoring of the infant's wellbeing cues through the mother's left visual and auditory fields, which project more directly to her right hemisphere, which in turn is more likely dominant for spatial abilities and emotion recognition from faces and speech (Hendriks et al., 2011; Malatesta et al., 2020a, 2021b). Accordingly, it has been demonstrated that the LCB is predicted by a typical right-hemispheric specialization for facial emotion processing (e.g., Harris et al., 2001, 2010; Bourne and Todd, 2004) and by a higher preference for the left profile of an infant face (which is considered more expressive compared with the right; Malatesta et al., 2020a, 2022), as well as by the cradler's socio-affective wellbeing (Weatherill et al., 2004; Reissland et al., 2009; Vauclair and Scola, 2009; Pileggi et al., 2015; Forrester et al., 2019; Malatesta et al., 2019a,b, 2021a). By reversing the perspective but remaining within the same conceptual framework, the LCB would in turn expose the more expressive side of the mother's face to the right hemisphere of the infant (i.e., their left visual and auditory fields), possibly canalizing the typical neurodevelopment of lateralized functions (Hendriks et al., 2011; Vervloed et al., 2011; Malatesta et al., 2020b, 2021b).

## Phylogenetic and ontogenetic factors involved in the left-cradling bias

The potential advantages of lateralization for the processing of emotions are in line with previous theoretical proposals (e.g., Vallortigara and Rogers, 2005, 2020) that functional hemispheric asymmetries might enhance cognitive efficiency (e.g., a positive association has been found between the typical leftward bias for emotional faces and better performance in emotion recognition; Workman et al., 2006; Watling and Bourne, 2013), and that the directional alignment of behavioral biases (at the population level) might respond to an evolutionary stable strategy molded by social pressures (e.g., the leftward bias for emotional faces would favor the monitoring of the dominant hand of others and their emotional states within the same hemisphere/perceptual field; Marzoli et al., 2014, 2022). Either point seems to be consistent with both phylogenetic and ontogenetic explanations. For instance, the significantly larger exposure to right-handed rather than left-handed individuals could entail—at either ontogenetic or phylogenetic levels, or both (e.g., see Marzoli et al., 2014, 2022; Lucafò et al., 2021 for similar considerations)—a perceptual and attentional bias toward the right side of others' body, broadly corresponding to a bias for the left visual field (from an allocentric perspective), as well as a leftward bias/right-hemispheric advantage for the processing of emotions from faces, which in turn could foster an overall dominance of the right hemisphere for emotions. Moreover, according to the phylogenetic view, the LCB might have evolved from the right-hemisphere dominance for emotion processing (e.g., see Palomero-Gallagher and Amunts, 2022) in the visual, auditory or tactile domains (Harris et al., 2001, 2010; Sieratzki and Woll, 2002; Bourne and Todd, 2004; Vauclair and Donnot, 2005; Donnot, 2007; Huggenberger et al., 2009). On the other hand, in line with the ontogenetic view, it has also been suggested that the mother's LCB preference might foster a (typical) leftward bias for emotional faces in the cradled individual (Vervloed et al., 2011). Analogously, some authors speculated that the LCB might account for the typical preference for left-facing profiles/cheeks, since when newborns are held on the mother's left arm during the first months of life, they are also exposed to her left profile during a critical period for the development of vision (McManus and Humphrey, 1973; Conesa et al., 1995; Conesa, 1996). In our opinion, these models are not mutually exclusive, indeed they all represent key mechanisms which should be extensively integrated within a broader framework of the environmental and genetic factors impacting functional brain lateralization in development and evolution.

## The left-cradling bias as a potential cue of fitness during evolution

As regards the emergence of the LCB, also the potential advantage for cradlers of not engaging their dominant arm/hand in other tasks cannot be excluded (van der Meer and Husby, 2006). In fact, it has also been proposed (Huheey, 1977) that the LCB might have emerged during human evolution for the very same advantage, which would be consistent with the notion that specific groups of genes have been selected in order to allocate different functions to different brain regions (e.g., emotion processing and cradling motor behavior in the right hemisphere, and limb motor control and language in the left hemisphere; Vingerhoets, 2019) in order to improve brain efficiency (e.g., by avoiding “cognitive crowding”; McManus, 2002; Lidzba et al., 2006; Nicholls et al., 2010; Papadatou-Pastou and Tomprou, 2015). And that is why we propose that the LCB may by right be included in a set of lateralized behaviors which can improve individuals' biological fitness. Although it is still unclear which are the evolutionary pressures underpinning behavioral asymmetries, animal studies suggest a common functional brain organization template in vertebrates. In this framework, the left hemisphere would be dominant for approach and manipulation processes, while the right hemisphere would be dominant for avoidance processes (i.e., detecting and reacting to threatening stimuli such as predators) and for monitoring individuals of the same species (including infants; see Vallortigara and Rogers, 2005 for a review). In fact, it has been proposed that the LCB would occur more likely when a face-to-face mother-infant interaction is underway (Vauclair and Donnot, 2005; Giljov et al., 2018). This would imply the advantage that socio-emotional information is mostly processed by the more specialized right hemisphere, as witnessed by several studies revealing a key role for emotional visual information in the LCB (Manning and Chamberlain, 1991; Harris et al., 2001, 2010; Bourne and Todd, 2004; Vauclair and Donnot, 2005; Huggenberger et al., 2009; Malatesta et al., 2020a, 2022).

## Environmental and genetic factors associated with asymmetries in mother-infant interaction

The LCB has been related to both mother's and infant's hand preferences in non-human primates (Hopkins et al., 1993; Manning and Denman, 1994; Hopkins, 2004; Boulinguez-Ambroise et al., 2022a,b), and the same has been suggested—at least to some extent—for humans (Dagenbach et al., 1988; van der Meer and Husby, 2006; Vauclair and Scola, 2009; Packheiser et al., 2019). Interestingly, some evidence of right-handedness at the population level observed in a number of primate species has been supposed to be due to intensive interactions with humans (Cochet and Byrne, 2013; Meguerditchian et al., 2013), and—as stated above—a likely role of social factors can be also hypothesized for the emergence of the LCB. In this regard, it should be noticed that many evolutionary scientists are attempting to establish common accounts for animal and human laterality. For instance, Boulinguez-Ambroise et al. (2022a) have recently contextualized the evolution of human handedness by analyzing limb preferences in animals, claiming that it would be related to both genetic and ontogenetic (including social interaction) factors, and that limb lateral preferences for actions directed either to self or to conspecifics (including cradling) is associated to hemispheric dominance for the processing of emotions. More generally, as regards the search for genetic factors of brain functional lateralization, single—or multiple—gene theories have been proposed to explain human handedness especially, and broad investigations in molecular genetics are still ongoing in order to identify the existence of specific loci (Cuellar-Partida et al., 2021). Although these studies seem to suggest a partially common ground among genetic variants affecting the development of brain functional lateralization and the occurrence of neurodevelopmental disorders (Wiberg et al., 2019), at present no single specific gene has yet been identified for either perceptual or motor asymmetries (e.g., the statistical frequencies of hand preference observed in families), which probably have a polygenic basis (Medland et al., 2009; McManus et al., 2013; Armour et al., 2014). In our opinion, the effects of epigenetic factors acting on the basis of genetically driven “core asymmetries” and of environmental influences should be taken into account when attempting to explain the origin of asymmetries in the processing of social stimuli and related behaviors (Marzoli et al., 2022). In this regard, it has not yet been studied which genetic and epigenetic factors affect the direction of cradling lateralization, nor which epigenetic changes can be induced by typical or atypical maternal cradling lateralization on the offspring. As suggested for the ontogenesis of handedness (for a review, see Schmitz et al., 2017), it is possible to hypothesize the involvement of several molecular processes underlying the epigenetic mechanisms modulated by environmental factors. In particular, DNA methylation, post-translational histone modifications and post-transcriptional regulation by non-coding microRNAs are currently among the most investigated epigenetic mechanisms in neuroscience, although their role in the development of functional brain lateralization has not been fully elucidated yet. Nevertheless, cradling laterality exhibits high heritability from generation to generation along the maternal line (Manning and Denman, 1994), but no genetic investigation has been carried out so far, and a crucial role for epigenetic factors cannot be ruled out. As far as we know regarding the effect of cradling laterality on the cradled individual, right-cradled infants have slightly higher odds of being left-handed at 19 months of age (Scola and Vauclair, 2010). Moreover, it has been shown that adults who had been cradled on the mother's right side during infancy showed a significant decrease of the left bias for emotional faces compared with individuals who had been cradled on the mother's left side (Vervloed et al., 2011). These findings seem to confirm our and others' proposal that maternal cradling lateral preferences might represent an important epigenetic factor in child neurodevelopment. In particular, it has been suggested—although not yet proven—that a reversed lateral holding position during infanthood might impair the information flow from faces (see also Hendriks et al., 2011), and thus the ability to perceive facial emotions and the development of socio-emotional competences later in life (Malatesta et al., 2020b,c, 2021b).

## Conclusion

It is plausible that lateralized cradling interactions might be part of a complex system involving several genetic and epigenetic mechanisms. It should be pointed out that, in line with this view, many ethological studies (mainly in avian species; e.g., Rogers, 1982, 1997) have shown interesting links between the exposure to lateralized environmental stimuli during the early stages of development and the later establishment of hemispheric asymmetries, which seem to entail the related appearance of specific behaviors (Nelson, 2022). In light of the literature reviewed above, we believe that the investigation of further possible effects of typical and atypical cradling on infant cognitive and affective development is warranted. In fact, whether the typical/atypical lateralization of cradling behavior is due to hereditary factors or is the outcome of maternal effects—or both—and what is its specific role within the broader framework of the development of human brain functional organization are fascinating but still pending questions.

## Author contributions

All authors listed have made a substantial, direct and intellectual contribution to the work, approved it for publication, and agree to be accountable for the content of the article.

## Conflict of interest

The authors declare that the research was conducted in the absence of any commercial or financial relationships that could be construed as a potential conflict of interest.

## Publisher's note

All claims expressed in this article are solely those of the authors and do not necessarily represent those of their affiliated organizations, or those of the publisher, the editors and the reviewers. Any product that may be evaluated in this article, or claim that may be made by its manufacturer, is not guaranteed or endorsed by the publisher.
